# Measles in Returning Adult Travelers

**DOI:** 10.4269/ajtmh.18-0053

**Published:** 2018-07

**Authors:** Christian Kositz, Werner C. Albrich

**Affiliations:** 1 Department of Internal Medicine, Cantonal Hospital St. Gallen, St. Gallen, Switzerland;; 2 Department of Infectious Diseases, Cantonal Hospital St. Gallen, St. Gallen, Switzerland

Measles is a highly contagious viral infection spread by droplets and commonly perceived as a childhood disease. Although vaccination is possible and proves to be very effective, a very high coverage is necessary to prevent outbreaks and to achieve elimination. The United States achieved this goal in 2000, however, cases of imported measles occur regularly.^1^ Although European countries had formerly achieved high vaccination coverage, in recent years coverage and therefore herd immunity has dropped mainly because of vaccine skepticism.^2^ The consequences have been regular outbreaks in groups who are opposed to vaccination and persons who depend on herd immunity.

This in turn increases the risk of introducing the infection into vulnerable populations by insufficiently vaccinated travelers who returned from countries where the disease is still endemic.^3^ Therefore, measles should be included in the differential diagnosis of fever and rash syndromes in returning travelers to avoid outbreaks and associated economic costs of outbreak control.^4^

The pictures show a 34-year-old returning traveler, who presented with measles and subsequently infected four more people, two of whom had a single vaccine and two relatives who were not vaccinated.

**Figure 1.**
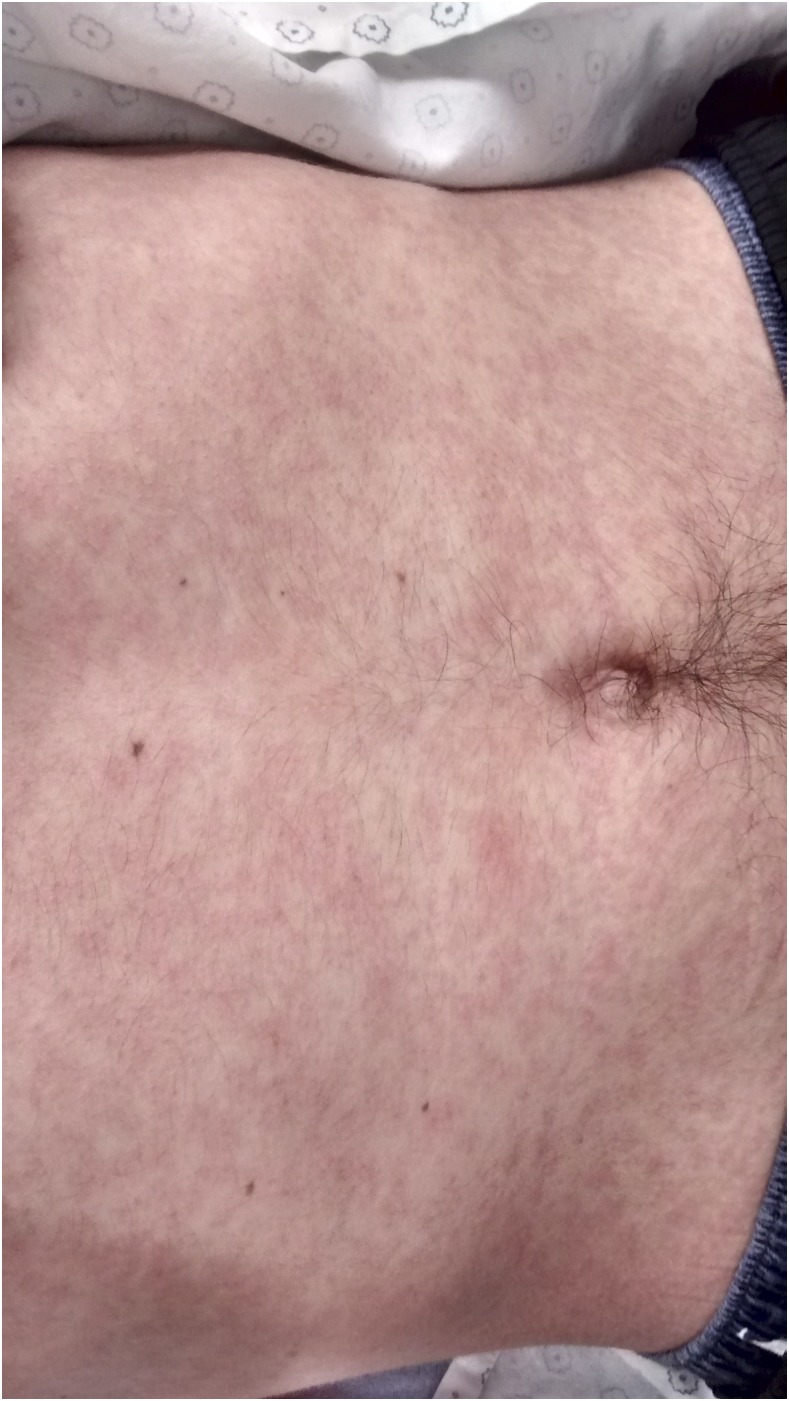
Morbilliform rash on the trunk. This figure appears in color at www.ajtmh.org.

**Figure 2.**
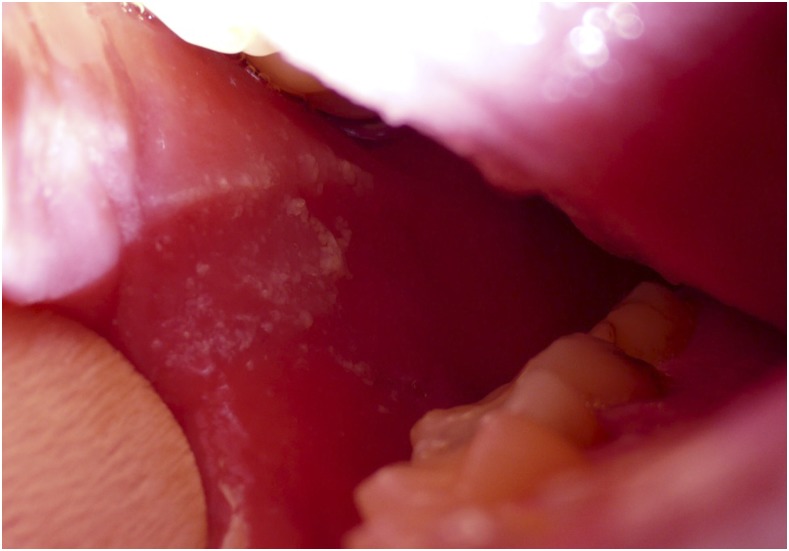
Koplik spots. This figure appears in color at www.ajtmh.org.

**Figure 3.**
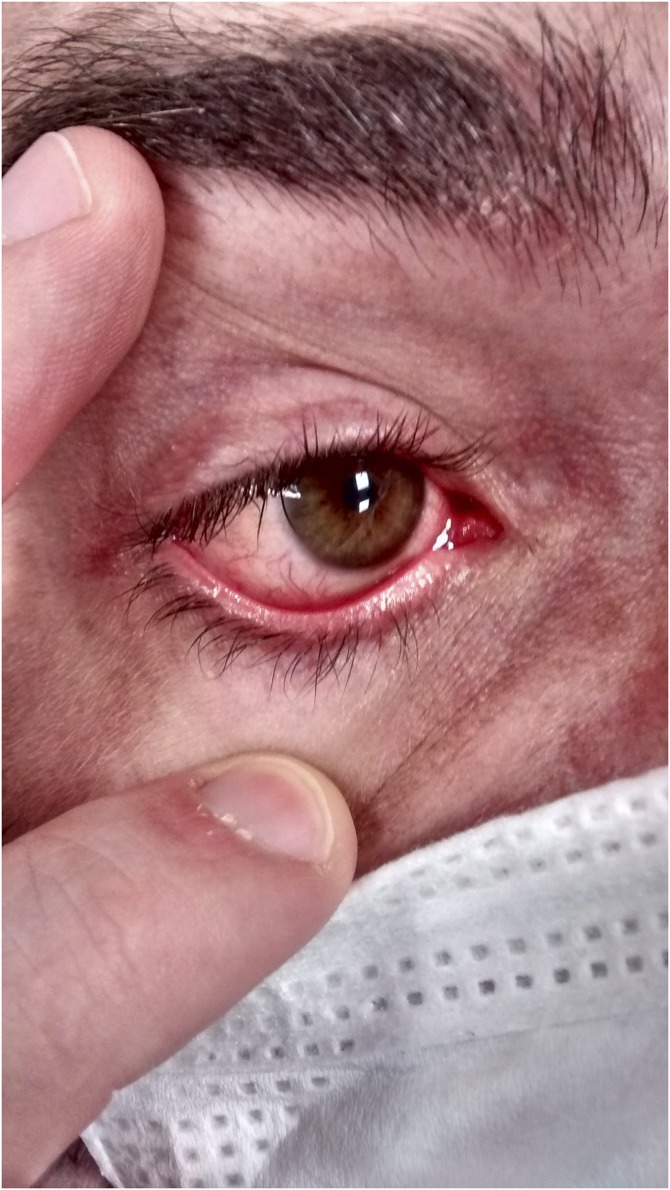
Conjunctivitis. This figure appears in color at www.ajtmh.org.

Typically presenting with a rash (Figure 1) that starts retroauricularly and spreads from face to trunk and extremities, other important features of measles are Koplik spots (Figure 2), which are pathognomonic, and conjunctivitis (Figure 3).
